# NF-κB Is Activated in CD4^+^ iNKT Cells by Sickle Cell Disease and Mediates Rapid Induction of Adenosine A_2A_ Receptors

**DOI:** 10.1371/journal.pone.0074664

**Published:** 2013-10-04

**Authors:** Gene Lin, Joshua J. Field, Jennifer C. Yu, Ruey Ken, Donna Neuberg, David G. Nathan, Joel Linden

**Affiliations:** 1 Division of Inflammation Biology, La Jolla Institute for Allergy and Immunology, La Jolla, California, United States of America; 2 Medical College of Wisconsin, Milwaukee, Wisconsin, United States of America; 3 Dana-Farber Cancer Institute, Boston, Massachusetts, United States of America; Wayne State University, United States of America

## Abstract

Reperfusion injury following tissue ischemia occurs as a consequence of vaso-occlusion that is initiated by activation of invariant natural killer T (iNKT) cells. Sickle cell disease (SDC) results in widely disseminated microvascular ischemia and reperfusion injury as a result of vaso-occlusion by rigid and adhesive sickle red blood cells. In mice, iNKT cell activation requires NF-κB signaling and can be inhibited by the activation of anti-inflammatory adenosine A_2A_ receptors (A_2A_Rs). Human iNKT cells are divided into subsets of CD4+ and CD4- cells. In this study we found that human CD4+ iNKT cells, but not CD4- cells undergo rapid NF-κB activation (phosphorylation of NF-κB on p65) and induction of A_2A_Rs (detected with a monoclonal antibody 7F6-G5-A2) during SCD painful vaso-occlusive crises. These findings indicate that SCD primarily activates the CD4+ subset of iNKT cells. Activation of NF-κB and induction of A_2A_Rs is concordant, i.e. only CD4+ iNKT cells with activated NF-κB expressed high levels of A_2A_Rs. iNKT cells that are not activated during pVOC express low levels of A_2A_R immunoreactivity. These finding suggest that A_2A_R transcription may be induced in CD4+ iNKT cells as a result of NF-κB activation in SCD. In order to test this hypothesis further we examined cultured human iNKT cells. In cultured cells, blockade of NF-κB with Bay 11–7082 or IKK inhibitor VII prevented rapid induction of A_2A_R mRNA and protein upon iNKT activation. In conclusion, NF-κB-mediated induction of A_2A_Rs in iNKT cells may serve as a counter-regulatory mechanism to limit the extent and duration of inflammatory immune responses. As activated iNKT cells express high levels of A_2A_Rs following their activation, they may become highly sensitive to inhibition by A_2A_R agonists.

## Introduction

Reperfusion injury following tissue ischemia is initiated by the activation of iNKT cells [1]–[3]. Widely disseminated ischemia-reperfusion injury is a manifestation of HbSS sickle cell disease that is caused by a homozygous point mutation in the ß-globin gene. The mutation promotes deoxyhemoglobin polymerization, formation of rigid sickled RBCs and production of large numbers of adhesive reticulocytes [4]. Tissue damaging vaso-occlusion in SCD has been viewed as resulting from obstruction of small blood vessels by sickled RBCs [5]. The clinical course of SCD is characterized by exacerbations that cause sudden painful vaso-occlusive crises (pVOC) and sometimes life-threatening episodes of acute chest syndrome (ACS). Recently, a modified paradigm has emerged suggesting that the clinical manifestations of SCD occur in part as a consequence of white cell activation [6]. As in ischemia-reperfusion injury, in NY1DD mice with SCD the activation of iNKT cells in response to tissue ischemia initiates an inflammatory cascade [7]. Poor lung function in SCD mice is ameliorated by iNKT cell depletion, by blockade of CD1d-restricted signaling [7], or by stimulation of anti-inflammatory A_2A_R receptors that are induced in SCD mice and that inhibit iNKT cell activation [8].

The A_2A_R is one of a family of four G protein coupled adenosine receptors (A_1_, A_2A_, A_2B_ and A_3_), that is expressed on most leukocytes and platelets and when activated exerts generally anti-inflammatory effects [9]. We have shown previously that pVOC in SCD patients results in the appearance of iNKT cells with high expression of activated NF-κB and cells that express high levels of anti-inflammatory A_2A_Rs. In prior studies we did not determine if the expression of activation markers occurs on the same or different cells than those that express high levels of A_2A_Rs. Since A_2A_R activation inhibits iNKT cell activation [10] we reasoned that the iNKT cells that are not activated may express high levels of A_2A_Rs. Here we demonstrate that NF-κB activation; T-bet induction, A_2A_R induction and cytokine production are all largely concordant (i.e. in the same cells) and occurs in a subset of CD4+ iNKT cells. The activation of cultured human iNKT cells results in induction of A_2A_R mRNA and protein expression that is blocked by NF-κB inhibitors. The findings suggest that A_2A_Rs are induced as a consequence of iNKT cell activation and may serve to limit the duration of their activation.

## Materials and Methods

All research involving human participants and the content of written informed consent forms were approved by the institutional review boards of the Medical College of Wisconsin and the La Jolla Institute for Allergy and Immunology. Consent forms signed by study participants are on file.

### Collection and processing of blood

Venous blood was obtained from adult patients, ages 18 to 60 years, with HbSS/HbSβ-thalassemia^0^ at Froedtert Hospital/Medical College of Wisconsin following informed consent. Paired samples separated by at least 30 days were collected from the same patient. Vaso-occlusive pain crisis was defined as an episode of pain related to SCD in the extremities, back, abdomen, chest or head lasting at least 2 hours and leading to a hospitalization [11]. Participants were determined to be at steady state when they were reporting no more than baseline pain and were at least 2 weeks from a hospitalization or emergency department visit for any reason.

### Flow Cytometry and statistics

RBCs in 0.3 ml blood were lysed (Biolegend) and remaining cells were washed with cold phosphate-buffered solution, pH 7.2 (PBS) containing 2 mM EDTA, resuspended in cold FACS staining buffer (PBS, 1%BSA, 1% human AB serum, 0.1% sodium azide) and incubated on ice for 10 minutes prior to staining. Remaining cells were incubated for 40 minutes at 4°C with fixable LIVE/DEAD stain to identify dead cells (Invitrogen) and then with fluorophore-conjugated antibodies directed against surface markers. Cells were washed twice with cold PBS, fixed, and resuspended in fixation/permeabilization buffer (BD biosciences) for 20 minutes at 4°C. After fixation, cells were washed twice with cold permeabilization buffer and incubated for 45 minutes at 4°C with fluorophore-conjugated antibodies specific for intracellular antigens. Cells were then washed with cold permeabilization buffer, fixed with 1% paraformaldehyde for 15 minutes at 4°C, washed with cold PBS, and resuspended in 0.3 ml of FACS staining buffer. The stained samples were stored at 4°C in the dark until flow cytometric analysis.

Invariant NKT cells were identified as live, CD19- (Invitrogen, SJ25-C1), CD3+ (Invitrogen & BD biosciences, UCHT1), and Valpha24-Jalpha18 TCR + (eBioscience, 6B11) cells and their CD4 phenotype was determined with anti-human CD4 antibody (BD biosciences, RPA-T4). For some experiments T cells were stained with biotinylated anti-Vα24 antibodies (Beckman Coulter IM2027)/brilliant violet streptavidin (Biolegend) that detect <1% of peripheral T cells. Staining with 6B11 identifies the iNKT subset of all Valpha24+ cells. Conventional T cells were identified as live, CD19-, CD3+, and 6B11- cells. The active phosphorylated form of p65 NF-κB was identified with anti-phospho-NF-κB p65 (Cell Signaling, 93H1). The human adenosine A_2A_ receptor was detected with anti-human receptor antibody 7F6-G5-A2 [12], [13] (Santa Cruz Biotech) conjugated to Alexa Fluor 647 (Invitrogen). IL-4, IFN-gamma, CD69 and the transcription factor T-bet were detected with anti-human IL-4 (BD, 8D4-8), anti human IFN-γ (eBioscience, 4S. B3) anti-human CD69 (BD, FN50) and anti mouse/human T-bet (BD, O4-46) antibodies, respectively. Flow cytometry was performed using a LSRII (BD biosciences) and data analysis performed using FlowJo software (Tree star). Data derived from patients sampled twice, once during pVOC and once at steady state were analyzed by the paired t-test.

### Human iNKT cell culture and activation

Human iNKT cell lines were generated from peripheral blood mononuclear cells (PBMCs) isolated from normal donor blood using a ficoll density gradient. A total of 120 million PBMCs were cultured for 12–14 days in culture medium (45% AIM V, Life Technologies, 50% RPMI 1640, Gibco, and 5% human AB serum, GemCell) supplemented with 100 IU/ml IL-2 (NCI) at and 100 ng/ml alpha-Galactosylceramide (alphaGalCer) (Funakoshi, KRN7000). Cells were stained with Live/Dead Aqua dye, anti-CD19, anti-CD3, and 6B11 antibodies and expanded iNKT cells were sorted using a BD FACSAria. iNKT cell lines were maintained with periodic restimulation by co-culturing a 1∶5 ratio of iNKT cells and γ-irradiated (4000 Rads) alphaGalCer (100 ng/ml) pulsed PBMCs. At the time of their use, iNKT cell lines were >97% pure as determined by flow cytometry with anti-CD3 and 6B11 antibodies. iNKT cells were incubated with vehicle, 1, 10 or 100 µM Bay 11–7082 ((E)-3-[(4-methylphenylsulfonyl]-2- propenenitrile), or 20 µM IKK inhibitor VII (Calbiochem) for 30 minutes prior to activation produced by seeding cells into wells coated with anti-CD3 antibody (1 μg/ml) (clone OKT3, eBioscience) or PBS and centrifuging them at 200× g for 2 minutes. At various time after activation, iNKT cells were harvested, immunostained to detect surface and intracellular markers, and then analyzed by flow cytometry.

### Quantitative real-time PCR

Purified human iNKT cells were harvested and lysed with RLT Plus lysis buffer. RNA was purified with Qiagen Allprep DNA/RNA Micro columns as described by manufacturer. cDNA was synthesized from RNA samples with a QuantiTect Reverse Transcription Kit as described by the manufacturer. Quantitative real-time PCR was performed using TaqMan Gene Expression assays and measured with a Roche 480 Light-Cycler. Relative RNA expression for A_2A_R, INFgamma, T-bet, and TNFalpha were normalized to RNA Polymerase IIA, set at 100.

## Results

### pVOC is associated with iNKT cell activation

Upon activation a subset of iNKT cells rapidly produce cytokines including INF-γ and IL-4 and begin to proliferate [14]. CD1d-restricted lipid antigens are presented to a subset of NKT cells that have receptors composed of an invariant Valpha14-Jalpha18 chain and a restricted repertoire of ß-chains [15]. iNKT cells in blood from SCD patients can be detected with fluorescent CD1d tetramers loaded with lipid antigens that bind to the invariant receptor. The human invariant receptor is also recognized by antibody 6B11 that binds to an invariant region on the Valpha14-Jalpha18 chain [16]. Pilot experiments using blood from controls and HbSS SCD patients demonstrate that 6B11 [16] can be used reliably to detect iNKT cells in SCD patients by flow cytometry.

**Figure 1:**
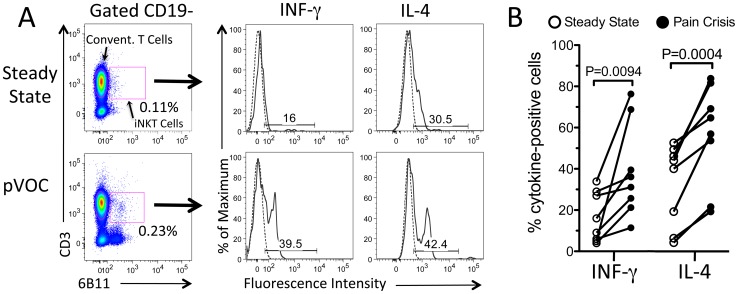
Painful vaso-occlusive crisis (pVOC) in SCD patients are associated with increases in the percentage of cytokine-positive iNKT cells. Blood was sampled from the same adult SCD patients during a pVOC and again at steady state. A) Immunostaining to detect iNKT cells and intracellular cytokines. iNKT cells (CD3+/6B11+, *solid lines*) and conventional T cells (CD3+/6B11-, *dotted lines*) in individual blood samples were identified by flow cytometry. The percentage of iNKT cells is calculated relative to the total CD3+ T cell population. B) Paired responses in 8 patients showing the percentage of iNKT cells that express high levels of IFN-gamma or IL-4 at steady state, during no more than typical pain, and during a pVOC. *P* values were calculated using one-tailed paired Student's T tests.

iNKT cell numbers are known to be increased in the blood of ambulatory patients with SCD compared to African American controls [7]. In the current study we examined for the first time paired blood samples taken at least four weeks apart from eight individual patients with HbSS, once during an acute pVOC, and once at steady state in the absence of more than typical pain. The mean age of patients providing paired samples was 27×8 years. With one exception, hydroxyurea was prescribed to all participants. Median time from hospital admission to sample collection during pVOC was 3 days. Consistent with expected clinical changes that occur during pVOC compared to steady state, patients showed increased pain scores (3 vs. 7, *P* = 0.01; 0 =  no pain to 10 =  worst pain) and decreased hemoglobin (9 vs. 7 g/dL, *P*<0.01) at the time of sample collection during pVOC. Figure 1A shows an example of the effects of pVOC on cells as assessed by flow cytometry. Cells that appear in the CD3+/6B11+ gate are defined as iNKT cells. We identified two distinct populations of iNKT cells in human blood with relatively low or high expression of IFN-gamma or IL-4. pVOC significantly increased the percentage of iNKT cells expressing high levels of both cytokines (Figure 1B). These findings confirm a characteristic feature of iNKT cells that distinguishes them from most conventional T cells; they produce both Th1 cytokines such as IFN-gamma, and Th2 cytokines such as IL-4 [17]. The findings demonstrate that in 8 of 8 patients examined acute pVOC was accompanied by a rapid increase in the percentage of iNKT cells in the circulation that are activated to produce cytokines.

### Enhanced concordant expression of activated NF-κB and A_2A_Rs in sickle iNKT cells

**Figure 2:**
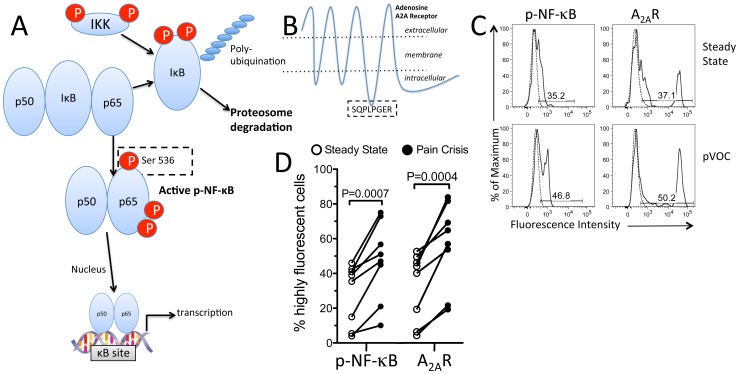
Pain crises in SCD patients cause activation of NF-κB and elevated expression of A_2A_Rs in circulating iNKT cells. A) Diagram depicting the molecular events leading to activation of NF-κB to regulate transcription. The dashed box depicts phospho-Ser536 on the p65 subunit of NF-κB (p-NF-κB) that is used as an activation marker. B) Diagram depicting the structure of the heptahelical A_2A_R located on the cell surface and the epitope on the third intracellular loop that is recognized by the 7F6-G5-A2 anti-A2AR monoclonal antibody. C) Flow cytometric analysis of p-NF-κB and the A_2A_R in iNKT cells (*solid lines*) and conventional T cells (*dashed lines*) of typical blood samples from a SCD subject at steady state and during a painful vaso-occlusive crisis. D) Paired responses in 8 patients showing the percentage of iNKT cells that have high p-NF-κB or A_2A_R expression at steady state and during a pain crisis. *P* values are based on one-tailed paired Student's T tests.

We next sought to determine if increased iNKT cell cytokine production in HbSS SCD patients occurs concordantly in cells with activated NF-κB, a known major proximal regulator of iNKT cell cytokine production [18]. We showed previously that on average, NF-κB is more activated in iNKT cells of patients during pVOC than in normal controls or in steady state SCD patients not experiencing pVOC [19]. As illustrated in Figure 2A, the activation of NF-κB is controlled by Iκ-kinase which catalyzes the rapid phosphorylation, ubiquination and proteolysis of the inhibitory subunit, IκB [20]. Once IκB dissociates, an active p50-p65 dimer of NF-κB can be phosphorylated at several sites, including Ser-526 of p65, and the dimer can translocate to the nucleus where it regulates transcription. An antibody that recognizes phospho-Ser-526 on p65 (p-NF-κB) was used to detect NF-κB activation by flow cytometry in iNKT cells. We also examined A_2A_R expression in single human iNKT cells as determined by immunofluorescence using a monoclonal antibody (7F6-G5-A2) that sensitively detects an epitope (SQPLPGER) localized to the A_2A_R third intracellular loop (Figure 2B). This was the most sensitive monoclonal antibody produced by immunization of mice with the purified full length recombinant human A_2A_R as the antigen, and has been used extensively for immunohistochemical localization of A_2A_Rs in the CNS [13], [21]. We showed previously that this Ab detects A_2A_R immunoreactivity in a permeabilized subset of cytokine-producing human CD3+ T cells, but not B cells [12]. In order to evaluate the ability of the antibody to detect A_2A_Rs in iNKT cells by flow cytometry, we first confirmed that it detects recombinant human A_2A_Rs stably transfected into HEK cells. The anti-A_2A_R antibody detected receptors in permeabilized, but not intact cells, consistent with localization of the antibody epitope on an intracellular-facing receptor domain (Figure 2B). Control experiments were performed to establish that storage and shipment of blood does not affect the expression in iNKT cells of p-NF-κB or the A_2A_R. We found that p-NF-κB and A_2A_R immunoreactivity are both elevated in iNKT cells of SCD patients during acute pVOC (Figure 2, C and D). The percentage of iNKT cells in the blood of steady state SCD patients expressing p-NF-κB or A_2A_Rs was highly variable, reflecting large differences among patients. However, all patients responded to a pVOC with increases in the percentages of iNKT cells expressing p-NF-κB and A_2A_Rs. The magnitude of the difference in anti-A_2A_R immunofluorescence intensity in activated vs. non-activated iNKT cells is well over 10-fold (Figure 2C), indicative of strong A_2A_R induction upon iNKT cell activation.

**Figure 3:**
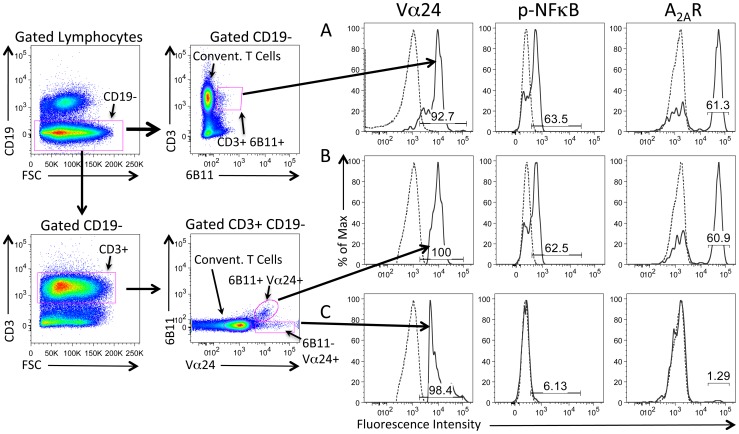
Sickle cell disease causes NF-κB activation and enhances A_2A_R expression only in 6B11+/Valpha24+ iNKT cells. A) iNKT cells in SCD patients identified as CD3+/6B11+ (*solid lines*) are positive for Valpha24 and partially positive for high p-NF-κB and high A_2A_R expression. Conventional 6B11- T cells are indicated with *dashed lines* in all panels. B,C) Among Valpha24+ T cells, only iNKT cells that are also positive for 6B11 express high levels of p-NF-κB and A_2A_Rs.

**Figure 4:**
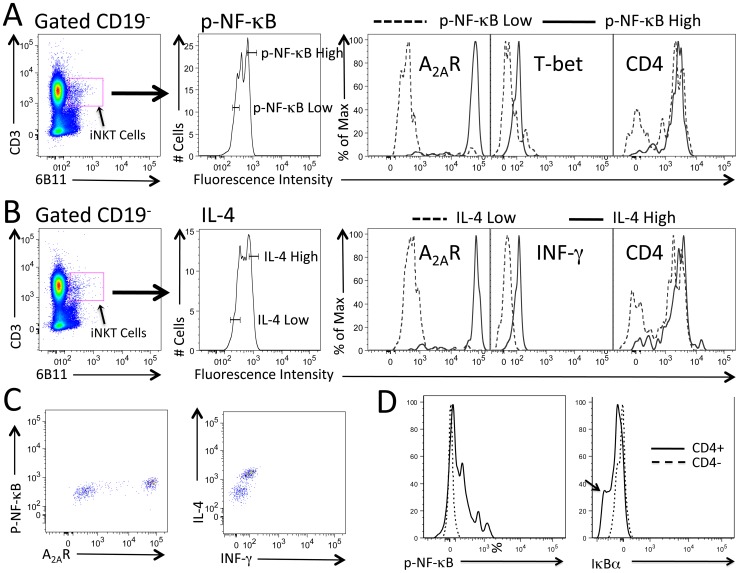
High concordance of cytokine, p-NF-κB and A_2A_R expression in iNKT cells from SCD patients during pVOC. iNKT cells (CD3+ 6B11+) were gated based on low or high expression of p-NF-κB or IL-4. A) High p-NF-κB expressing iNKT cells (*solid lines*) are associated with high immunostaining for the A_2A_R, T-bet and CD4. B) High IL-4 expressing iNKT cells are associated with high immunostaining for the A_2A_R, IFN-gamma and CD4. C) Dot plots illustrate that most cells dually stained for p-NF-κB and A_2A_Rs are either double positive or double negative. Most cells dually stained for IL-4 and IFN-gamma are either double positive or double negative. D) Among iNKT cells, only the CD4+ subsets are activated. The same subset of CD4+ iNKT cells that express high levels of p-NF-κB also express low levels of IκBalpha (*arrow*).

Among lymphocytes in the blood of SCD patients, only iNKT cells express high levels of p-NF-κB and A_2A_R immunoreactivity whereas conventional T cells express only low levels (Figures 2). T cells that are Valpha24+ include all iNKT cells as well as a small fraction of conventional T cells. A comparison of Valpha24+ iNKT cells that are positive for 6B11 (Figure 3) with the subset of conventional T cells that are positive for Valpha24+ but negative for 6B11 confirms that even among Valpha24+ T cells, only the subset of 6B11+ cells express high levels of p-NF-κB and A_2A_Rs. These findings support the conclusion that tissue injury in SCD generates lipid antigens that are uniquely capable of activating the invariant TCRs found on iNKT cells but not conventional T cells. Figure 4 A,B shows high concordance among iNKT cells that express high levels of IL-4, A_2A_Rs and IFN-gamma. Concordance between p-NF-κB and A_2A_Rs and between IL-4 and IFN-gamma is also illustrated in the dot plots of Figure 4C which reveal predominantly cells that are double negative or douple positive for p-NF-κB/A_2A_R or IL-4/IFN-gamma, single positive iNKT cells are rarely observed. These findings suggest that A_2A_Rs are induced as a consequence of iNKT cell activation.

NF-κB activation is triggered by the degradation of the IκB inhibitory subunit (Figure 2A). As expected, an increase in p-NF-κB was associated with a decrease in IκB expression in activated iNKT cells (Figure 4D). Among circulating iNKT cells, most are CD4+, but some are CD4-. We noticed that iNKT cells that are activated by SCD (p-NF-κB-high and IκB-low) are also CD4+; thus CD4+ iNKT cells appear to be particularly sensitive to activation by SCD (Figure 4D).

**Figure 5:**
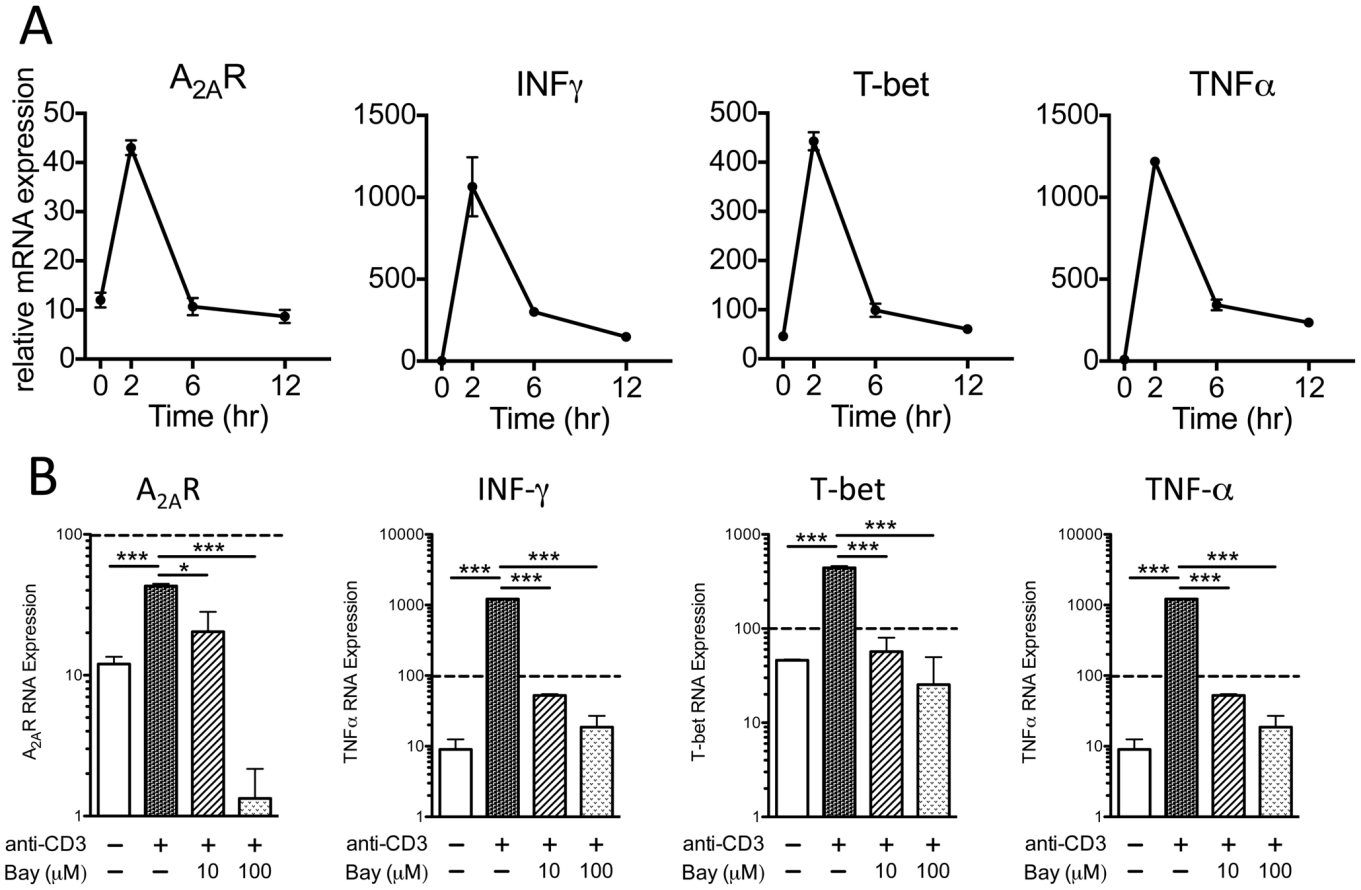
Induction of transcripts for the A_2A_R, INF-gamma, T-bet and TNFalpha in activated human iNKT cells is decreased by the NF-κB inhibitor Bay 11 –**7082 (Bay).** (A) Time course of expression of mRNAs for the A_2A_R, INF-gamma, T-bet, and TNFalpha following activation of cultured human iNKT cells. (B) Relative mRNA at 2 hours in iNKT cells preincubated for 30 minutes with 0, 10 or 100 µM of Bay. The dashed line in each plot designates expression of RNA Polymerase IIA. *P* values were calculated by ANOVA and Dunnett's multiple comparison test, N = 3. * P<0.05, ** P<0.01, *** P<0.001. The results are typical of duplicate experiments.

In order to determine if activation of human iNKT cells rapidly induces A_2A_R mRNA and protein we expanded human iNKT cells in culture. After 13 days in culture, reactivation of these cells by plate-bound anti-CD3 antibody produced a rapid transient induction of A_2A_R mRNA, as well as transcripts for the Th1 transcription factor, T-bet, and Th1 cytokines IFN-gamma and TNFalpha (Figure 5A). The induction of A_2A_R mRNA and other factors were inhibited by pretreating cells for 30 min before their activation with the NF-κB inhibitor Bay 11–7082 (Figure 5B). Inhibition of A_2A_R mRNA production at 2 hours by Bay 11–7082 suggests that NF-κB is a direct activator of A_2A_R transcription.

**Figure 6:**
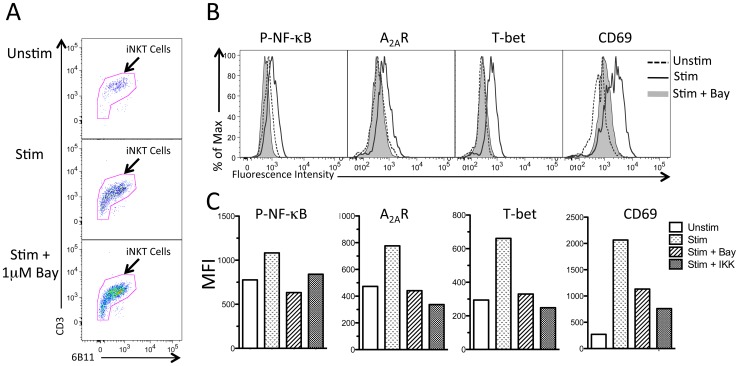
Increase in protein immunoreactivity of phospho-NF-κB (p65), A_2A_R, T-bet and CD69 upon activation of human iNKT cells is attenuated by NF-κB inhibitors Bay 11 –**7082 (Bay, 1** **µM) or IKK inhibitor VII (IKK, 20** **µM).** Cultured human iNKT cells were incubated with vehicle or NF-κB inhibitors for 30 minutes prior to activation with plate-bound anti-CD3 antibody or PBS. Following incubation for 24 hours, iNKT cells were harvested, immunostained to detect surface and intracellular markers, then analyzed by flow cytometry. (A) Expression on stimulated or unstimulated iNKT cells of CD3 and 6B11, used as markers of iNKT cells. (B) Fluorescence intensity of unstimulated and stimulated iNKT cells cultured in the absence or presence of 1 μM Bay. (C) Mean fluorescence intensity (MFI) of phospho-NF-κB, A_2A_R, T-bet, and CD69 in unstimulated and stimulated iNKT cells cultured in the absence and presence of 1 µM Bay or 20 µM IKK inhibitor 7. The results of typical of triplicate experiments.

We next examined the effects of NF-κB inhibitors on the expression of A_2A_R protein (immunoreactivity) and other activation markers on cultured human iNKT cells as determined by FACS immunofluorescence. Activation of iNKT cells for 24 h with plate-bound anti-CD3 antibody resulted in the appearance of some iNKT cells with low expression of CD3 and the invariant receptors recognized by 6B11 (Figure 6A), probably due to down-regulation of these molecules. Activation also resulted in production of an increase in the fluorescence intensity on iNKT cells of antibodies detecting phospho-NF-κB, A_2A_Rs, T-bet and CD69 (Figure 6B) that was prevented by pretreatment of cells with 1 μM Bay 11–7082 or 20 μM IKK inhibitor VII (Figure 6C). The doses shown were selected on the basis of pilot dose-ranging experiments. Excessively high doses of NF-κB inhibitors resulted in iNKT cell apoptosis. The findings suggest that the expression of the A_2A_R as well as T-bet and CD69 all depend on transcription that is controlled by NF-κB.

## Discussion

iNKT cells are activated by ischemia-reperfusion injury of liver [10], heart [22], [23] kidney [3]. Recent mouse studies revealed that generalized inflammation in SCD also is precipitated in large part by the activation of CD1d-restricted iNKT cells [7]. These data suggest that ischemic tissue injury as a result of pVOC triggers sterile activation of innate immunity that is propagated by activation of iNKT cells [10]. Consistent with these findings, previous studies have demonstrate that in addition to RBC pathology, SCD is associated with white cell and platelet activation that contribute to vascular inflammation and vaso-occlusion [24]–[27]. It has not been clear how this inflammation is initiated or propagated to different cell types. The findings of the current study indicate that pVOC in SCD patients is consistently associated with rapid iNKT cell activation. Since A_2A_R activation inhibits iNKT cell activation [10], [19] we reasoned that iNKT cells with high A_2A_R expression would be resistant to activation. Contrary to this expectation, we found a high degree of overlap between NF-κB activation and high A_2A_R expression in individual iNKT cells. These finding suggest that A_2A_Rs are elevated as a consequence of iNKT cells activation, and may serve to inhibit their activation over time.

In people, iNKT cells are divided into CD4+ and CD4- (primarily CD4/CD8 double negative) characterized as Th0/helper and Th1/effector phenotypes, respectively [28], [29]. The data show that CD4+ iNKT cells of the helper phenotype are preferentially activated as a result of SCD. This may occur because CD4 engagement by co-receptors on APCs potentiates iNKT cell activation [30]. The findings suggest that the activation of invariant TCRs by host antigens requires CD4 co-stimulation to preferentially activate the CD4+ subset of iNKT cells. Activation of CD4+ iNKT cells results in phosphorylation on Ser-536 of the p65 subunit of NF-κB and transcription of IFN-gamma and IL-4. Prior studies in mice indicated that NF-κB plays an essential role in the activation of iNKT cells; iNKT cell ontogeny and activation requires signal processing by NF-κB [18]. The administration of NF-κB inhibitors to SCD mice has been shown to prevent ischemia/reperfusion-mediated activation of mononuclear and endothelial cells [31]. In the current study we show that although a variable percentage of iNKT cells in the circulation of SCD patients are activated at steady state, the activated percentage was increased in 8 of 8 patients during pVOC. These findings suggest that as in mice, the CD1d-restricted NF-κB-dependent activation of iNKT cells in SCD patients orchestrates an inflammatory cascade that contributes to pVOC and acute chest syndrome.

CD1d-restricted activation of iNKT cells can occur in response to lipid antigens that are produced by various pathogens. However, CD1d-restricted iNKT cell activation may be triggered by autologous host lipid antigens such as ß-D-glucopyranosylceramide [32]. Once activated, iNKT cells produce IFN-γ that can stimulate parenchymal cells to produce IFN-gamma-inducible chemokines that are chemotactic to other leukocytes [7]. IFN-gamma also stimulates APCs to enhance the release of cytokines such as IL-12 and IL-18 that can directly amplify iNKT cell activation [33]. Even weak TCR-mediated activation sensitizes iNKT cells to these cytokines [34]. Inflammation and RBC-medicated vaso-occlusion may trans-activate platelets and neutrophils [27] to propagate additional inflammation and vaso-occlusion. Other disease processes that cause ischemic tissue injury may also produce rapid iNKT cell activation noted here during acute pVOC of SCD. Theses include other vaso-occlusive diseases such as myocardial infarction and stroke, tissue transplantation and peripheral vascular disease.

The activated fraction of human iNKT cells was found to express much higher A_2A_R immunoreactivity than the non-activated fraction of iNKT cells or conventional T cells [13], [21]. High A_2A_R immunoreactivity was also found on a small percentage of iNKT cells in SCD blood at steady state, but this percentage is significantly increased in iNKT cells of SCD patients during pVOC. Hence, like p-NF-κB, A_2A_R immunoreactivity is a biomarker of iNKT cell activation. As with human neutrophils [35] and macrophages [36], the effect of A_2A_R activation on T cells [37]–[39] and iNKT cells [10] is to inhibit inflammation predominantly by elevating cyclic AMP and activating protein kinase A. This counteracts NF-κB activation in part by inhibiting proximal events involved in TCR-mediated signaling transduction [40]. In the case of macrophages, blockade of NF-κB downstream of TLR stimulation has been shown to attenuate the induction of A_2A_Rs [36]. In the current study we found a high concordance among iNKT cells expressing high levels of p-NF-κB and high levels of the A_2A_R. This is consistent with the idea that A_2A_R induction may be downstream of NF-κB activation and serves as a counter-regulatory mechanism to limit inflammation. We also found that other activation markers are concordant with NF-κB activation and high A_2A_R expression in iNKT cells. These include IFN-gamma, the cardinal Th1 inflammatory cytokine, T-bet, the master Th1 transcription factor and the Th2 cytokine, IL-4. The production of IL-4 may be significant because it could contribute to airway hypersensitivity responses that are common in SCD children [41]–[43]. In order to confirm a role for NF-κB in regulating A_2A_R transcription, we demonstrated that NF-κB inhibitors prevent induction of A_2A_R mRNA expression and A_2A_R receptor expression (as estimated by immunofluorescence) upon activation of cultured human iNKT cells.

## Conclusions

The results of this study suggest that A_2A_Rs are strongly induced as a result of iNKT cell activation. Similar induction of A_2A_Rs has been noted after activation of macrophages by endotoxin [36] or activation of conventional T cells [39], [44]. Activation of cultured human iNKT cells was found to produce a rapid induction of A_2A_R mRNA and protein that could be blocked by inhibitors of NF-κB. An increase in receptor expression is known to increase the functional potency of agonists [45]. These findings suggest that induction of A_2A_R receptor expression is downstream of NF-κB activation, and that A_2A_R induction may be generally used by immune cells to limit the extent and duration of inflammatory responses.
