# Correction: NF-κB Is Activated in CD4^+^ iNKT Cells by Sickle Cell Disease and Mediates Rapid Induction of Adenosine A_2A_ Receptors

**DOI:** 10.1371/journal.pone.0117760

**Published:** 2015-02-06

**Authors:** The PLOS ONE Staff

The Competing Interests section is incorrect. The Competing Interests section should read: Joshua Field, M.D., M.S., Joel Linden, Ph.D. and David Nathan, M.D. each have consulting agreements with NKT Therapeutics, Inc. Joel Linden, Ph.D. is an inventor on a patent issued to the University of Virginia, which claims the use of adenosine A2A agonists for the treatment of sickle cell disease; he owns shares in Adenosine Therapeutics LLC, which licensed this patent. This does not alter our adherence to all the PLOS ONE policies on sharing data and materials.
